# Difficulties in retrieving specific details of autobiographical memories and imagining positive future events in individuals with acute but not remitted anorexia nervosa

**DOI:** 10.1186/s40337-022-00684-w

**Published:** 2022-11-18

**Authors:** Johanna Louise Keeler, Georgia Peters-Gill, Janet Treasure, Hubertus Himmerich, Kate Tchanturia, Valentina Cardi

**Affiliations:** 1 Section of Eating Disorders, Department of Psychological Medicine, Institute of Psychiatry, Psychology and Neuroscience, King’s College London, 103 Denmark Hill, London, SE5 8AF UK; 2 School of Psychology, University of Exeter, Exeter, Devon, UK; 3 South London and Maudsley NHS Foundation Trust, Bethlem Royal Hospital, Monks Orchard Road, Beckenham, Kent, BR3 3BX UK; 4 Illia State University, Tbilisi, Georgia; 5 Psychological Set Research and Correction Center, Tbilisi State Medical University, Tbilisi, Georgia; 6 Department of General Psychology, University of Padova, Padua, Italy

**Keywords:** Anorexia nervosa, Autobiographical memory, Depression, Eating disorders, Episodic future thinking, Memory

## Abstract

**Introduction:**

The factors that contribute to the maintenance of anorexia nervosa (AN) are not fully understood, although it is generally accepted that depression is a core feature and contributes to poor prognosis. Individuals with depression tend to have difficulties in producing specific details of autobiographical memories and future episodes. Our aim was to investigate autobiographical memory and episodic future thinking (EFT) in individuals with AN (n = 46), people recovered from AN (recAN; n = 40), and non-affected controls (n = 35).

**Method:**

Using a remotely administered computerised version of the autobiographical memory test and episodic future thinking task, we measured six aspects of memory retrieval and EFT generation: specificity, detailedness, difficulty in remembering/imagining, positivity, vividness and realism. Memory and EFT cue valence was manipulated; cues were either positive, neutral, or disorder-related/negative. As the production of EFTs is theoretically linked to the ability to retrieve autobiographical memories, the relationship between autobiographical memory specificity and EFT specificity was explored. To investigate whether autobiographical memory and EFT performance were independent of performance on other forms of cognition, working memory, verbal fluency and cognitive flexibility were measured.

**Results:**

People with AN had difficulties retrieving specific details of autobiographical memories and rated autobiographical memories as less positive overall, and less vivid when primed by positive cues. People with a lifetime diagnosis (currently ill or recovered) reported greater difficulty in retrieving memories. The AN group generated less positive EFTs, particularly to positive and neutral cues. Comorbid depressive symptoms had some contribution to the observed findings. Lastly, in all groups autobiographical memory specificity predicted EFT specificity.

**Discussion:**

Problems with retrieving specific details of autobiographical memories and simulating positive EFTs may be a state feature of AN. Treatments targeted at alleviating depressive symptoms, as well those targeted towards facilitating memory retrieval or reconsolidation, and the construction of positive EFTs, may contribute to hope for recovery and strengthen the sense of self beyond the disorder.

**Supplementary Information:**

The online version contains supplementary material available at 10.1186/s40337-022-00684-w.

## Plain English summary

Problems with generating future episodic thoughts are a feature of depression, and depression is commonly present in individuals with anorexia nervosa (AN). In this study, the ability to remember autobiographical events and to think about events in the future was measured using a standardised task, in a group of 46 individuals with AN, 40 individuals recovered from AN and 35 individuals without AN. The results showed that individuals with AN showed difficulties in recalling specific details of autobiographical memories, and rated them as less positive and vivid. People with current AN or recovered from AN reported greater difficulty in recalling autobiographical memories. Lastly, people with AN produced less positive episodic future events. Comorbid depressive symptoms had some contribution to these difficulties. This study suggests that individuals with AN have difficulty recalling specific details of autobiographical memory and generating positive episodic future thoughts. This is likely to cast a negative bias on their view of the future, which could lessen hope for recovery.

**Supplementary Information:**

The online version contains supplementary material available at 10.1186/s40337-022-00684-w.

## Introduction

Anorexia nervosa (AN) is characterised by a persistent state of malnutrition and is often associated with cognitive and emotional difficulties [1, 2]. Features include low cognitive flexibility [3–5], a tendency to focus on details [6] and high levels of negative affect and anxiety [7]. Over time, these issues may develop into neuroprogressive features which may or may not recover with weight restoration [8–12].

Processes that sit at the interface between cognitive and emotional processing are of particular interest and yet are surprisingly understudied. Examples of such processes are autobiographical memory and episodic future thinking (EFT). These are influenced by both the integrity of allied cognitive functions, such as cognitive flexibility and attention to details, and negative emotions, which might create a bias towards recalling or generating negative events. These processes are also closely tied together, and it is proposed that EFT abilities are linked with the integrity of autobiographical memories. For example, the “constructive episodic simulation hypothesis” posits that details from past events are flexibly recombined to simulate novel future events [13]. EFT facilitates several processes, including far-sighted decision making, spatial navigation, emotional regulation, prospective memory, planning and self/identity formation [14].

The few studies in the literature which have characterised autobiographical memory or episodic future thinking in eating disorders have demonstrated that individuals with AN show impairments in autobiographical memory, such as a tendency to overgeneralise past memories with difficulties in recalling specific details [15, 16]. Furthermore, individuals with AN can have a bias towards negative salient information and disorder-relevant information [17–19]. In line with the theoretical predictions about the association between autobiographical memory and episodic future thinking, individuals with AN have also shown difficulties in simulating specific details of future events [20], and a bias towards simulating negative future events [21]. However, no studies have examined the relationship between autobiographical memory retrieval and EFT production in AN or have examined the two together using comparable tasks. It is also unclear whether these difficulties are related to the specific valence/content of the cues (e.g. positive, negative, disorder-relevant) or whether they are present regardless of the cue’s valence. Finally, the literature in populations recovered from AN is sparse; it is unclear whether these difficulties persist after recovery or are “state” features.

In healthy individuals, the intensity of positive memories tends to persist over time, whereas negative memories fade, meaning that positive memories are more salient. This is known as the “fading affect bias”, which begins within 12 h of an event and persists for at least three months [22], and is thought to help with maintaining psychological wellbeing [23]. In individuals with depressive disorders, this bias is reduced, suggesting that the intensity of negative memories persists over time in the same way as positive memories [24, 25]. Affective disorders such as major depressive disorder are commonly comorbid with AN, in approximately half of cases [26]. Moreover, neuroprogressive changes over the course of the illness, together with the psychosocial toll of living with AN may beget or exacerbate depressive symptoms [27]. Meta-analytic findings indicate reduced autobiographical memory and EFT specificity in patients with major depressive disorder [28–30]. Therefore, it may be the case that symptoms of depression contribute to or drive problems with autobiographical memory retrieval and EFT in individuals with AN. Relatedly, it could also be the case that these problems constitute neuroprogressive sequelae of the illness, which are not yet fully understood [27].

The aim of the study was to investigate autobiographical memory retrieval and EFT in individuals with AN compared with unaffected controls and individuals recovered from AN. Specifically, we were interested in whether autobiographical memory recall and future event construction is altered depending on the valence of the cue (i.e. positive, negative disorder-relevant, or neutral). Moreover, we were interested in testing the constructive episodic simulation hypothesis [13] by investigating whether the ability to construct specific EFTs was related to the specificity of autobiographical memory. Our main hypothesis was that individuals with acute AN would be worse at recalling specific details of autobiographical memories and producing details of EFTs compared with controls and individuals recovered from AN, and that these problems would be greater for positive and neutral cues, compared with disorder-relevant cues. We also expected the ability to produce specific details of autobiographical memories to predict the ability to produce specific details of EFT in all groups, as posited by the “constructive episodic simulation” hypothesis. Finally, we expected that problems with autobiographical memory/EFT generation would be independent of problems with general cognition, and would be partially related to depressive symptomatology.

## Methods

### Participants and design

This study followed a cross-sectional, between-groups design. A total of 121 participants took part in this study. Participants with acute AN (acAN; n = 46; AN-restrictive = 31; AN-binge-purge = 7) were recruited from the South London and Maudsley NHS trust, email research circulars at King’s College London, recruitment websites (e.g. www.beateatingdisorders.org.uk) and social media. Healthy controls (n = 35) and participants recovered from AN (recAN; n = 40; AN-restrictive = 36; AN-binge-purge = 1) were recruited from email circulars, recruitment websites and social media.

Participants with acAN had to have a diagnosis of anorexia nervosa from a clinician (i.e. according to the Diagnostic and Statistical Manual of Mental Disorders—5th edition; DSM-5, [31], or International Classification of Diseases; ICD, [32]) and a body mass index (BMI) ≤ 18.5 kg/m^2^. Participants in the recAN group had to have a (self-reported) historical lifetime diagnosis of anorexia nervosa from a clinician, with no current behavioural or psychological symptoms (Eating Disorder Examination Questionnaire (EDE-Q; [33]) clinical cut off Global Score of ≤ 3) and a BMI of over 18.5 kg/m^2^ for ≥ 1 year. The clinical cut-off of ≤ 3 on the EDE-Q was deemed as appropriate by the research team, given a cut-off of ≥ 4 is commonly used as an indicator of clinical significance in the literature [34]. Diagnoses of AN in the acAN and recAN groups were verbally confirmed by participants during a pre-study phone call where a full clinical history was taken. Healthy controls had to have no self-reported current, or history of, any psychiatric or neurological disorders, and a current BMI of ≥ 18.5 kg/m^2^. For all groups, participants had to be aged > 18 years; be fluent in the English language; have access to a computer and stable internet connection; have no uncorrected visual or auditory impairments; and have no aphantasia as assessed by the Vividness of Visual Imagery Questionnaire [35]. Additionally, all participants were required to have no history of or current post-traumatic stress disorder, substance abuse or psychotic disorder, as these psychiatric conditions are associated with difficulties with autobiographical memory retrieval [36–38] but have a relatively low prevalence in individuals with AN compared to other psychiatric conditions such as affective and anxiety disorders [26, 39].

### Measures

#### Demographic and clinical variables

##### Demographics

A bespoke demographic questionnaire measured the following variables: age, ethnicity, gender, years of education, highest level of education obtained and medication usage. Self-reported weight and height was recorded, which was used to calculate BMI in kg/m^2^.

##### Eating disorder psychopathology and characteristics

The EDE-Q [33] was used to quantify eating disorder psychopathology (Cronbach’s alpha = 0.97). The EDE-Q is a 28-item self-report questionnaire that assessed the severity and features associated with eating disorders, and constitutes four subscales (Eating Concern, Shape Concern, Weight Concern, Restraint), which are averaged to provide a Global score. Participants in the recAN and acAN groups were asked to estimate their lowest BMI and the duration of their eating disorder diagnosis (in years), as well as to self-report their AN subtype (based on criteria, which were provided). Participants in the recAN group were asked to estimate how long they had been recovered for (in years).

##### Comorbidities

Participants were asked to self-report whether they have received a diagnosis of a comorbid psychiatric disorder, including mood disorders, anxiety disorders, personality disorders or any others. Symptoms of depression and stress were measured using the Depression, Anxiety and Stress Scale-21 (DASS-21) [40], which is a 21-item self-report questionnaire assessing depression, anxiety and stress (7 items each) over the previous week using a series of statements and responses ranging from 0 (“Did not apply to me at all) to 3 (“Applied to me very much or most of the time”). The Cronbach’s alpha for the DASS-21 was 0.95. Anxiety was measured using the DASS-21 anxiety subscale, and the Generalised Anxiety Disorder Assessment (GAD-7; [41], which is a 7-item scale measuring symptoms of anxiety over the previous 2 weeks (Cronbach’s alpha = 0.93).

##### Sleep quality and sleepiness

Poor sleep quality and duration has been found to interfere with autobiographical memory retrieval [42, 43], which may be a potential confound. To assess sleep quality, participants were asked to score their sleep on a visual analogue scale ranging from 0 (“terrible”) to 100 (“excellent”). Participants were also asked to specify how long they slept in hours over the previous three nights, which was used to compute an average. Sleepiness was measured using the Epworth Sleepiness Scale [44], which is an 8-item questionnaire recording participants’ general tendency to doze in a variety of different situations, ranging from 0 (“no chance of dozing”) to 3 (“high chance of dozing”). The Cronbach’s alpha was 0.82. Scores of 10 and above are indicative of excessive sleepiness.

#### Autobiographical memory and future thinking

The Autobiographical Memory Test (AMT; [45]) involves the presentation of a series of cue words (e.g. sad), to which participants are asked to describe a memory. For episodic future thinking, a written version of the EFT variant of the AMT was used (the EFT task; EFT-T), which has an identical procedure but with the instruction to simulate an EFT rather than recall a memory [46].

In this study, participants completed a written version of the task where they are given two minutes to respond. A total of nine word cues of differing valence (positive, neutral and negative/eating disorder-relevant) were given. Instructions given at the beginning of the task aligned with the standardised instructions of the AMT [45] and EFT-T [47] and included the following: the memory should be a specific, personal experience of the participant that lasted no longer than a day; the future event should be a hypothetical or likely event that they would be personally involved in; both memories and future events should be inspired by or directly related to the cue word. Participants were instructed to consider as many details of the memory/event as possible (e.g. what is being done, who they are with, feelings and emotions). Participants were also instructed that a different memory should be used for each cue, although no restrictions on the time frame were made. Participants were given two examples of responses to word cues.

Words presented to participants consisted of 18 cues, including six neutral (e.g. “house”, “book”), six positive (“excited”, “relaxed”) and six disorder-relevant negative (“stigma”, “hunger”) cues. Participants in the control group saw negative cues rather than disorder-relevant, to ensure that the valence and arousal of the cues was consistent between groups. These cues were determined ahead of the study in collaboration with individuals who have lived experience of AN (n = 6), and with healthy controls (n = 16). These groups rated the cues in terms of valence and arousal, which were matched across groups. Nine of these cues elicited past events (List A) and nine elicited future events (List B), which were counter-balanced between participants. Participants completed the nine memory trials before the nine future event trials. Cues were presented in a fixed order: neutral, positive, then disorder-relevant (or negative). Participants were given a 30 s rest break between trials.

The main dependent variable was the specificity of the response. Responses were blindly coded independently by two researchers (J.K. and G.P.G.), using the a manual obtained from the creator of the EFT task [47], based on their level of specificity: 1 = a specific event that occurred/could occur, contained within 24 h and located in a time and place; 2 = an extended memory/future thought, occurring over a period of longer than 24 h; 3 = a categoric memory/future thought, i.e. referred to as an event that has occurred repeatedly; 4 = a semantic associate; or 5 = an off-task response. Additional dependent variables were the participant ratings of how positive, detailed, realistic, vivid and difficult to remember/imagine the memory/future event was. For these variables, participants were asked to rate their answer on a series of 7-point Likert scales (1 = not at all, 7 = very much). Therefore, AMT and EFT-T outcome variables included: the researcher-rated specificity (scored 0–4), and five participant-rated measures: positivity, detail, realism, vividness and difficulty to remember (scored 0–7).

#### Neuropsychological assessment

Cognitive abilities that are likely to interfere with, or facilitate, memory retrieval and EFT construction include working memory abilities, cognitive flexibility and verbal fluency. Working memory was assessed as poor working memory shows a strong association with the overgeneralisation of autobiographical memories [48]. Verbal fluency was assessed as the (in)ability to generate words may interfere with the ability to write detailed accounts of memories and/or future episodes [49]. Lastly, cognitive flexibility was assessed as the ability to generate EFTs is hypothesised to be reliant on the flexible recombination of details from autobiographical memories [13].

##### Working memory

The digit span forward and backward [50] is a measure of short term and working verbal memory. It is thought that the forward task measures working memory and attention, whereas the backward task also tests cognitive control and executive function. In the forward version of this task, participants are shown a string of numbers (e.g. 2, 4, 9, 6) for 500 ms (with a 250 ms fixation length) and are then asked to recall the number string (e.g. 2496). If a participant gets two consecutive trials correct, the number string increases in length from a minimum of two digits up to a maximum of eight digits. In the backward version, number strings are presented in an identical format, but participants are required to write the number strings backwards (e.g. 6942). Performance on this task was computed as span scores (the maximum number of digits correctly produced).

##### Verbal fluency

The Word Fluency Task is a written task designed to assess verbal generational abilities. This is an adaptation of the Controlled Oral Word Association Test [51]. Participants are asked to write as many words as they can that start with the letter “F” for two minutes. Following this, they then write as many words as they can that start with the letter “A” for two minutes, and then the letter “S”. The total number of correct words and total number of errors (e.g. perseveration) are the main outcome measures.

##### Cognitive flexibility

The Wisconsin Card Sorting Test (WCST) [52] is the most widely used measure of cognitive flexibility in the eating disorders literature. This study utilised a shortened computerised version, which comprises of 64 trials, rather than 128 trials, administered remotely via computer. Participants are required to sort cards according to implicit principles (i.e. colour, shape and number). The rule by which participants are to sort the cards is changed after every 10 consecutive correct responses, which is unannounced. The main outcome measures include: the number of categories achieved, the percentage of perseverative errors (continuing to respond the same despite rule change) and number of correct answers.

### Procedure

This study used the online platforms Gorilla [53], Inquisit [54] and Qualtrics (www.qualtrics.com) to create and host the tasks. Interested participants were sent a bespoke eligibility screening questionnaire via Qualtrics. If eligible, participants provided informed consent and received a phone call from a researcher where they were instructed on how to optimise the study environment, such as: turning their mobile phones off, making sure they are in a quiet space with minimal distractions, putting the browser on full-screen, etc. Researchers were able to monitor participants’ progress in the study, live, through the web interface. The study took the form of two online testing sessions taking up to an hour each, spaced 24 h apart at approximately the same time of day. A researcher was available during the study sessions over email and telephone, in the case that participants need the task instructions to be explained in more detail. Prior to the first session, participants were sent a link to complete a battery of questionnaires.

The tasks in the first session included the Word Fluency Test, followed by the AMT and EFT-T. At the end of the AMT and EFT-T, a short positive mood induction (3 min) of relaxing music was administered. During the second session, participants completed the digit span (forward and backward), the emotional mnemonic similarity task (not presented here; [55]), a prospective memory task (not presented here) and the WCST [52]. Throughout both sessions, participants were given optional 2-min rest breaks between tasks.

Informed consent was obtained from all participants using an approved participant information sheet and consent form, and the study received ethical approval from the Camden and King’s Cross Research Ethics Committee (REF: 21/LO/0338). Participants were reimbursed for their time.

### Statistical analysis

#### Power analysis

Effect sizes taken from a previous meta-analysis [28] suggest an effect size of Hedges *g* =  − 0.84 for episodic future thinking impairments in those with a psychiatric diagnosis. Similarly, studies using the original AMT to assess autobiographical memory in AN have found medium-large effect sizes [15]. A power calculation using GPower [56] indicated that to detect a medium-large difference in our EFT outcome using a 4-group (for a larger study with a binge-type eating disorder group) analysis of variance (ANOVA) (α = 0.05, β = 0.95), a total sample size of 104 would be required.

#### Main analyses

All analyses were conducted using SPSS [57]. Baseline homogeneity of demographic and clinical characteristics was assessed using Chi-square test statistics (for categorical data) and ANOVAs (for continuous data). Individual analyses of covariance (ANCOVA) models were run using the AMT and EFT-T outcomes as dependent variables, within a 3 (cue valence: negative, neutral, positive) × 3 (group: control, acAN, recAN) model, with age and years of education as covariates. Sensitivity analyses (see Additional file 1: Tables S4 and S5) were run in order to examine whether controlling for ethnicity, DASS-Depression scores or average sleep over the last 3 days would alter the results, where significant effects had been found. For the exploratory neuropsychological outcomes, ANCOVA models were run using the task performance as a dependent variable, group as a fixed factor, with the aforementioned covariates.

A linear regression model was run using the following procedure, in each group individually: firstly, AMT specificity was entered as an initial block (Model 1), using the “Enter” procedure, followed by control regressors (age, years of education) in a second block (Model 2). Regression models also examined the effect of DASS-Depression on AMT and EFT-T task outcomes within the AN group. Separate linear regression models using the “Enter” procedure included DASS-Depression, age and years of education as regressors within one block, using the six AMT and EFT-T outcomes as dependent variables. See Additional file 1: Table S6 for the full results of these analyses.

The *p* < 0.05 threshold of significance was utilised for all analyses, and effect sizes are reported in the form of Cohen’s *d* and standardised *ß* in the case of regression models*.* All post-hoc group-comparisons were Bonferroni corrected.

## Results

### Demographic and clinical characteristics

Age and ethnicity differed between groups (see Table 1). The recAN group was of a lower age than the acAN (*p* = 0.005) and control (*p* = 0.031) groups. There was greater ethnic diversity in the control and recAN groups compared with acAN. As expected, the use of psychotropic medication differed significantly between the groups, with more participants in the acAN and recAN groups reporting medication use. However, the numbers using hormonal contraceptives were similar across groups (see Table 1). Self-reported BMI significantly differed between groups, with the control group reporting a higher BMI than recAN (*p* < 0.001) and acAN (*p* < 0.001), and recAN higher than acAN (*p* < 0.001). Additionally, eating disorder psychopathology, depression, anxiety and stress differed between groups, with acAN scoring higher on all measures than both recAN and controls (all *p* < 0.05), and recAN scoring higher than controls (all *p* < 0.05). Participants with acAN reported worse sleep quality compared with recAN (*p* = 0.019) and controls (*p* < 0.001). The recAN participants also reported worse sleep quality compared with controls (*p* = 0.040). The acAN group reported a lower duration of sleep over the past 3 nights than recAN (*p* = 0.043) and controls (*p* = 0.012). Despite this, sleepiness measured by the Epworth Sleepiness Scale was similar between groups.

**Table 1:** Comparison of demographic and clinical characteristics between controls (HC), the acute anorexia nervosa (acAN) group and recovered AN group (recAN)

	HC (n = 35)	acAN (n = 46)	recAN (n = 40)	*F-*value (df) or χ2 (df)	*p *value (Cohen’s *d*)
*Demographic characteristics*					
Age, years M ± SD	26.6 ± 10.0	27.4 ± 7.2	22.9 ± 4.2	4.46 (2,118)	0.014* (0.55)
Gender, n (%)				5.02 (2,118)	0.081 (0.42)
Female	35 (100%)	43 (94%)	40 (100%)		
Non-binary	0 (0%)	3 (6%)	0 (0%)		
BMI, kg/m^2^ M ± SD	23.7 ± 4.5	16.2 ± 2.5	20.8 ± 1.9	62.04 (8)	< 0.001** (2.05)
Ethnicity, n (%)					
White	23 (68%)	43 (94%)	32 (80%)	19.85 (8)	0.011* (0.89)
Black	2 (6%)	0 (0%)	0 (0%)		
Asian	7 (20%)	0 (0%)	4 (10%)		
Multiracial	2 (6%)	3 (6%)	2 (5%		
Arab	0 (0%)	0 (0%)	2 (5%)		
Years of education, M ± SD	17.5 ± 2.6	16.6 ± 2.2	16.3 ± 2.0	2.54 (2,118)	0.083 (0.41)
*Clinical characteristics*					
Use of psychotropic medication, n (%)					
Anti-depressants	0 (0%)	19 (41%)	10 (25%)	19.42 (2)	< 0.001 (0.91)**
Mood stabilisers	0 (0%)	1 (2%)	1 (3%)	1.05 (2)	0.590 (0.18)
Anti-psychotics	0 (0%)	7 (15%)	0 (0%)	13.51 (2)	0.001 (0.68)**
Anti-anxiety	0 (0%)	4 (9%)	1 (3%)	4.88 (2)	0.087 (0.40)
Uses hormonal contraceptives, n (%)	11 (32%)	9 (21%)	6 (17%)	2.54 (2)	0.282 (0.28)
Sleep quality (VAS), M ± SD	75.8 ± 18.3	52.9 ± 24.8	64.8 ± 24.4	9.86 (2,118)	< 0.001** (0.82)
Average sleep last 3 days, hours M ± SD	7.5 ± 1.0	6.7 ± 1.7	7.3 ± 1.2	3.76 (2,118)	0.026* (0.51)
Epworth Sleepiness Score, M ± SD	5.4 ± 3.8	6.3 ± 4.7	5.3 ± 5.0	0.65 (2,118)	0.526 (0.21)
EDE-Q Global, M ± SD	0.8 ± 1.0	3.6 ± 1.3	1.7 ± 1.2	61.25 (2,118)	< 0.001** (2.04)
DASS Depression, M ± SD	5.0 ± 6.6	19.4 ± 12.3	11.0 ± 9.4	21.56 (2,118)	< 0.001** (1.21)
DASS Anxiety, M ± SD	4.6 ± 6.0	14.2 ± 9.3	9.1 ± 7.7	14.68 (2,118)	< 0.001** (1.00)
DASS Stress, M ± SD	7.7 ± 7.3	23.5 ± 10.0	14.7 ± 8.3	33.10 (2,118)	< 0.001** (1.50)
GAD-7, M ± SD	3.2 ± 3.6	12.7 ± 5.4	8.7 ± 5.5	35.49 (2,118)	< 0.001** (1.55)

*acAN* acute anorexia nervosa, *BMI* body mass index, *DASS* Depression, Anxiety and Stress Scale, *EDE-Q.* Eating Disorder Examination Questionnaire, *GAD-7* Generalised Anxiety Disorder scale, *HC* healthy controls, *M* mean, *ns* non-significant, *recAN* recovered from anorexia nervosa, *SD* standard deviation, *VAS* visual analogue scale

^*^Significant at the *p* < 0.05 threshold, **significant at the *p* < 0.01 threshold. All post-hoc analyses were Bonferroni corrected

A total of 35 (76%) participants with acAN reported being amenorrhoeic (i.e. reported missing periods in the last 3–4 months). Within the recAN group, the average recovery duration in years was 2.2 ± 1.4, ranging between 1 and 6 years (reported by n = 33 participants). The estimated lowest BMI was significantly lower in acAN (M ± SD = 13.6 ± 2.2) than recAN (M ± SD = 15.1 ± 1.8; *p* = 0.062; *d* = 0.69). The duration of diagnosis was similar between the acAN group (M ± SD = 8.7 ± 6.5 years) and the recAN group (M ± SD = 6.5 ± 3.5 years; *p* = 0.053; *d* = 0.42). The most common reported comorbid diagnosis was an affective disorder (acAN = 23 (50%), recAN = 15 (38%)), followed by anxiety disorders (acAN = 18 (39%), recAN = 16 (40%)), personality disorder (acAN = 4 (9%)), post-traumatic stress disorder (acAN = 3 (7%), recAN = 1 (3%)) and neurodevelopmental disorders (e.g. attention deficit hyperactivity disorder, autism spectrum disorder; acAN = 1 (2%), recAN = 1 (3%)).

### Autobiographical memory and episodic future thinking

**Fig. 1:**
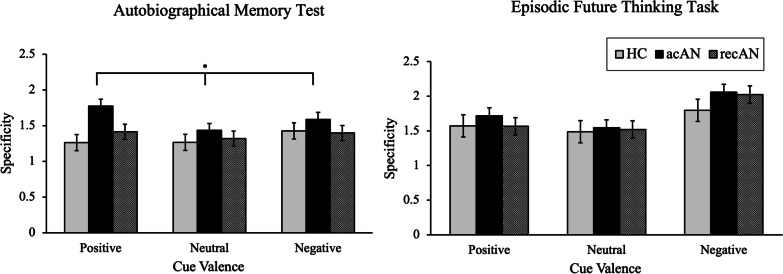
Schematic of between-group differences in autobiographical memory and episodic future thinking (researcher-rated) specificity, by cue type. Specificity was inversely scored with higher scores representing less specificity. Error bars represent standard errors. *Significant at *p* < 0.05

**Fig. 2:**
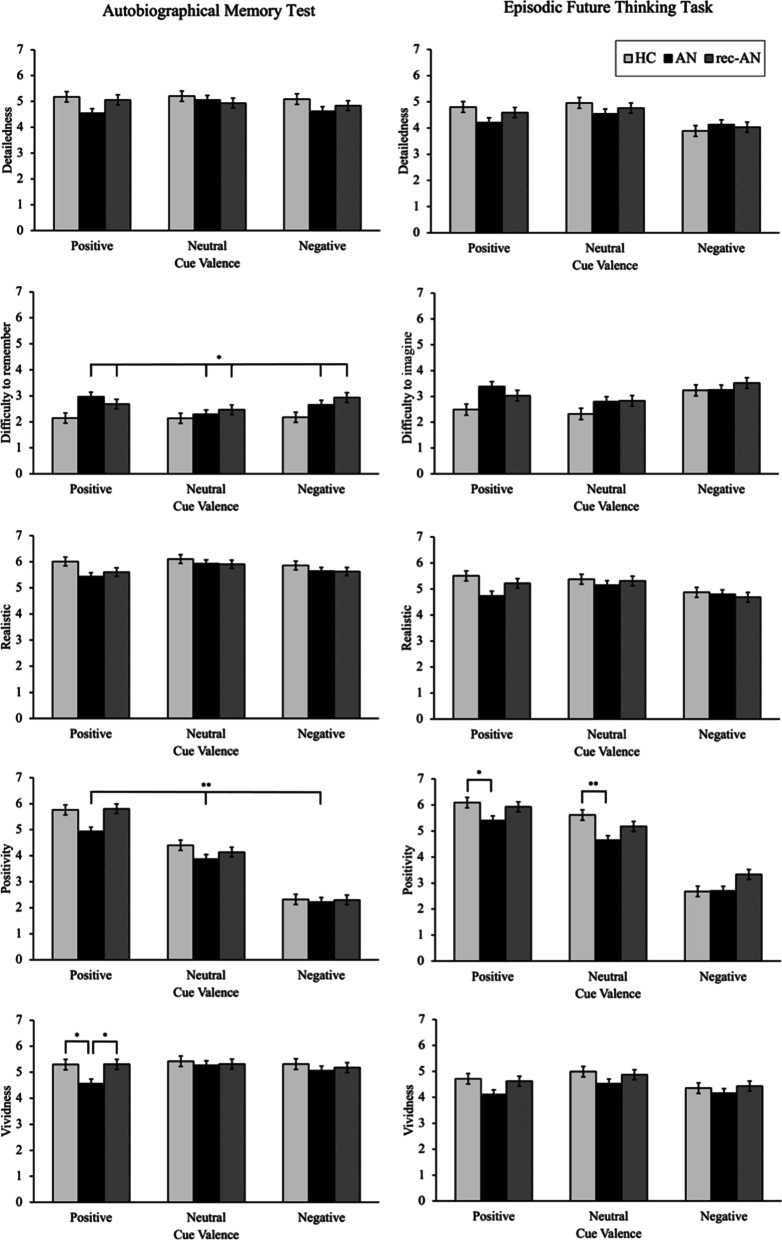
Schematic of between-group differences in autobiographical memory and episodic future thinking (participant-rated) detailedness, difficulty to remember, realistic-ness, positivity and vividness, by cue type. Error bars represent standard errors. **Significant at *p* < 0.01, *significant at *p* < 0.05

See Table 2 for the main effects of valence, group and valence x group for the autobiographical memory test and episodic future thinking task, and Figs. 1 and 2 for schematics of the differences between groups for researcher-rated outcomes (i.e. specificity) and participant-rated outcomes (i.e. detailedness, difficulty to remember, realistic-ness, positivity and vividness), respectively. The effects of the covariates (age, education) on the cue valence x group interaction term are presented in Additional file 1: Table S2, and the effects of the covariates on the between-group effect are presented in Additional file 1: Table S3.

**Table 2:** Effect of cue valence, group and cue valence x group in ANCOVA models for autobiographical memory and episodic future thinking outcomes

	Valence		Group		Valence × Group	
	F-value (*df* = 2,232)	*p* value (Cohen’s *d*)	F-value (*df* = 2,116)	*p* value (Cohen’s *d*)	F-value (*df* = 4,232)	*p *value (Cohen’s *d*)
*Autobiographical memory task*						
Specificity^a^	0.62	0.542 (0.14)	3.59	0.031* (0.50)	1.68	0.155 (0.34)
Detailedness^b^	0.12	0.890 (0.06)	2.08	0.129 (0.38)	1.65	0.163 (0.34)
Difficulty to remember^b^	0.33	0.720 (0.11)	4.55	0.013* (0.56)	1.94	0.104 (0.36)
Realistic^b^	0.65	0.524 (0.16)	1.91	0.153 (0.36)	1.09	0.360 (0.28)
Positivity^b^	4.48	0.012* (0.39)	6.44	0.002** (0.67)	2.02	0.093 (0.38)
Vividness^b^	2.42	0.091 (0.29)	1.84	0.163 (0.36)	2.54	0.040* (0.42)
*Episodic future thinking task*						
Specificity^a^	0.12	0.886 (0.06)	0.57	0.568 (0.20)	0.61	0.656 (0.20)
Detailedness^b^	0.08	0.926 (0.06)	0.73	0.485 (0.22)	2.38	0.053 (0.40)
Difficulty to imagine^b^	0.46	0.634 (0.13)	2.66	0.075 (0.43)	1.91	0.110 (0.36)
Realistic^b^	2.61	0.076 (0.30)	1.65	0.196 (0.34)	2.00	0.095 (0.37)
Positivity^b^	1.89	0.154 (0.26)	6.57	0.002** (0.67)	3.33	0.011** (0.48)
Vividness^b^	0.64	0.478 (0.16)	2.14	0.123 (0.39)	0.95	0.413 (0.26)

**Significant at *p* < 0.01, *significant at *p* < 0.05. All analyses were run with age and years of education as covariates

^a^Experimenter rated, ^b^Participant rated

In the autobiographical memory test, a significant interaction between group and valence emerged for memory vividness only (F(4,232) = 2.54; *p* = 0.040; *d* = 0.42). Post-hoc comparisons revealed that in response to positive cues, participants with acAN reported less vivid memories than controls (*p* = 0.032; 95% CI [0.05, 1.41]) and recAN (*p* = 0.025; 95% CI [− 1.41, − 0.07]). The group x valence interaction for memory vividness was non-significant when additionally controlling for ethnicity (F(2,228) = 2.26; *p* = 0.063; *d* = 0.40), DASS-Depression scores (F(2,230) = 1.53; *p* = 0.193; *d* = 0.33) or average sleep over the past three days (F(2,228) = 2.36; *p* = 0.054; *d* = 0.41) (see Additional file 1: Table S4).

The effect of group was significant for memory specificity (F(2,116) = 3.59; *p* = 0.031; *d* = 0.50). Overall, individuals with acAN produced less specific memories, regardless of cue valence, compared with controls (*p* = 0.044; 95% CI [− 0.56, − 0.01]). Moreover, the effect of group was significant for difficulty in remembering (F(2,116) = 4.55; *p* = 0.013; *d* = 0.56) and positivity (F(2,116) = 6.44; *p* = 0.002; *d* = 0.67). Individuals with acAN produced less positive memories regardless of cue valence compared with controls (*p* = 0.004; 95% CI [0.12, 0.85]) and recAN (*p* = 0.020; 95% CI [− 0.76, − 0.05]). Both the acAN (*p* = 0.035; 95% CI [− 0.94, − 0.03]) and recAN (*p* = 0.022; 95% CI [− 1.02, − 0.06]) groups reported more difficulty in remembering autobiographical memories than controls. When additionally controlling for DASS-Depression scores, the group effects on memory specificity (F(2,115) = 1.63; *p* = 0.201; *d* = 0.33) and difficulty in remembering (F(2,115) = 2.92; *p* = 0.058; *d* = 0.45) were non-significant, although controlling for average sleep and ethnicity did not change the results (see Additional file 1: Table S4).

A significant interaction between group and valence emerged for the positivity of constructed future events (F(4,232) = 3.33; *p* = 0.011; *d* = 0.48). Post-hoc comparisons revealed that in response to positive (*p* = 0.012; 95% CI [0.12, 1.26]) and neutral cues (*p* = 0.003; 95% CI [0.28, 1.66]), the acAN group rated EFTs as less positive than controls. No other significant main or interaction effects were found. The group x valence interaction for the positivity of future events was non-significant when additionally controlling for DASS-Depression scores (F(2,230) = 2.33; *p* = 0.057; *d* = 0.40). Controlling for ethnicity or average sleep did not change the results (see Additional file 1: Table S5).

### The effect of AMT specificity on EFT-T specificity

Overall, all models significantly predicted EFT-T specificity, both with and without the inclusion of age and years of education as regressors, for all groups (see Table 3). In healthy controls, AMT specificity accounted for 20% of variance in EFT-T specificity (F(1,34) = 9.51; *p* = 0.004). In acAN, AMT specificity accounted for 24.6% of variance in EFT-T specificity (F(1,45) = 15.69; *p* < 0.001). In recAN, AMT specificity accounted for 17.1% of variance in EFT-T specificity (F(1,39) = 9.03; *p* = 0.005). In all groups, the inclusion of age and education did not significantly contribute to the predictive value of the models (all *p* > 0.05).

**Table 3:** Results of linear regression models investigating the effect of autobiographical memory specificity (Model 1) and control regressors (age and years of education; Model 2) on the specificity of episodic future thoughts, stratified by group

Model	Adjusted R^2^ (SE)	Independent Variable	Unstandardised Beta (SE)	*ß*	T	*p* value
*Healthy controls*						
1	0.200 (0.41)	(Constant)	0.988 (0.216)		4.581	< 0.001**
		AMT specificity	0.478 (0.155)	0.473	3.084	0.004**
2	0.233 (0.40)	(Constant)	0.304 (0.486)		0.625	0.537
		AMT specificity	0.424 (0.155)	0.419	2.734	0.010*
		Age	0.009 (0.007)	0.204	1.337	0.191
		Years of education	0.029 (0.027)	0.169	1.097	0.281
*Acute anorexia nervosa*						
1	0.246 (0.69)	(Constant)	0.711 (0.296)		2.405	0.020*
		AMT specificity	0.679 (0.171)	0.513	3.960	< 0.001**
2	0.269 (0.68)	(Constant)	1.621 (0.860)		1.884	0.066
		AMT specificity	0.563 (0.182)	0.425	3.084	0.004**
		Age	0.020 (0.015)	0.179	1.290	0.204
		Years of education	− 0.076 (0.047)	− 0.213	− 1.598	0.118
*Recovered anorexia nervosa*						
1	0.171 (0.56)	(Constant)	0.795 (0.300)		2.648	0.012*
		AMT specificity	0.638 (0.213)	0.438	3.004	0.005**
2	0.149 (0.56)	(Constant)	0.389 (0.896)		0.433	0.667
		AMT specificity	0.670 (0.218)	0.460	3.068	0.004**
		Age	0.023 (0.022)	0.156	1.008	0.320
		Years of education	− 0.009 (0.047)	− 0.031	− 0.199	0.834

*AMT* autobiographical memory test, *ß* standardised beta, *SE* standard error

**Significant at *p* < 0.01, *significant at *p* < 0.05

### The effect of depression on AMT and EFT-T outcomes in the AN group

Whilst entering age and years of education as control regressors, DASS-Depression only predicted EFT specificity in the acAN group, with the overall model accounting for 18.9% of variance (F(3,45) = 4.49; *p* = 0.008). Higher levels of depression were a positive predictor of specificity scores (N.B. these were inversely scored with higher scores representing less specificity; therefore higher depression predicted lower specificity of EFTs) (*ß* = 0.298; *t* = 2.103; *p* = 0.042). See Additional file 1: Table S6 for the full results of the regression models.

### Exploratory cognitive outcomes

Additional file 1: Table S1 displays the statistical results of the exploratory cognitive outcomes. Performance on the cognitive tasks was largely the same between groups, with only two aspects of the Wisconsin Card Sorting Task differing between groups: the number of trials to complete the first category (F(2,111) = 3.83; *p* = 0.025; *d* = 0.53) and “Learning to Learn” (F(2,111) = 3.37; *p* = 0.038; *d* = 0.49), whereby the acAN group scored lower than the recAN group (p = 0.010, p = 0.012, respectively).

## Discussion

The present study aimed to investigate autobiographical memory retrieval and episodic future thinking in people with acute AN and those recovered from AN. Overall, our first hypothesis was partially confirmed, in that the acAN showed a state-like reduction in the ability to retrieve specific details of autobiographical memories (i.e. performed worse than controls and recAN). However, their ability to produce specific details of EFTs was intact. The deficit in producing specific details of autobiographical memories was pervasive regardless of cue type, thus not supporting our second hypothesis, which predicted the deficit would be greater for memories primed by positive and neutral cues. Our third hypothesis based on the constructive episodic simulation hypothesis [13] predicted that the specificity of EFTs would be predicted by autobiographical memory specificity. This was confirmed; autobiographical memory specificity did predict EFT specificity in all groups, although as aforementioned, individuals with acAN surprisingly did not show a deficit in producing specific details of EFTs.

The present study yielded a number of additional findings. For example, individuals with acAN also rated memories as less positive and more difficult to remember, and rated memories primed by positive cues as less vivid, compared with controls and recAN. EFTs primed by positive and neutral cues were rated as less positive in the acAN group compared with controls. Notably, controlling for between-group differences in depressive symptoms rendered most of the aforementioned findings non-significant, although individuals with acAN still produced less positive autobiographical memories when controlling for depressive symptoms. However, within the acAN group, depressive symptomatology only predicted the ability to produce specific details of EFTs, but did not predict any outcome of autobiographical memory retrieval or any additional EFT outcomes. The recAN group showed no impairments in autobiographical memory retrieval and EFT construction, other than reporting greater difficulty in remembering than controls, implying that the aforementioned findings in the acAN group may be state features of AN. It should be noted that despite persisting depression in recAN, they performed well on the autobiographical memory and EFT tasks, which may imply the existence of a protective factor present in individuals recovered from AN.

Overall, the acAN group recalled less specific memories, regardless of cue type. This aligns with previous research that has found deficits in the recall of specific autobiographical memories [58, 59], across cue types [16], in individuals with AN. This also aligns with findings in populations with depressive disorders [60]. The acAN group rated memories recalled in response to a positive cue as less vivid, implying a level of bias in the quality of their memory recall. Individuals with depressive disorders show a reduced “fading affect bias” [24], which pertains to the reduction of intensity of negative memories over time in relation to positive memories. Aligning with the literature in depressive disorders, it is possible that negative (negative-disorder-related) memories also remain more salient over time in individuals with acAN. However, this may be even more amplified in AN, whereby positive memories become even less salient over time in comparison to negative-disorder-related or neutral memories. It is possible that this leads to more negative constructions of EFTs as was exhibited in our study. However, it should be noted that vividness is likely to be a related, but distinct, construct compared to emotional intensity, which is typically the measure used to calculate the fading affect bias [24]. Future studies should endeavour to investigate the nature and quality of this possible affective bias in memory intensity and its relation to EFT in individuals with AN.

It is generally accepted that low mood is a common component of the clinical presentation of the acute stages of AN [7], which is thought to be a sequalae of, or exaggerated by, undernutrition. However, depression may also be involved in the pathogenesis of AN [61, 62]. Our results are consistent with a contribution of depression to the aforementioned autobiographical memory and EFT difficulties in AN. However, our finding that depression only predicted one EFT outcome (i.e. specificity; and did not predict any autobiographical memory outcome) does not align with this supposition. This, together with our finding that the ability to produce specific EFT was preserved in AN (which is usually found to be reduced in depression; [28]) reflects the complex relationship between AN and depressive symptoms. This latter finding somewhat contrasts with other previous findings in AN populations [20]. For example, Rasmussen and colleagues found that in comparison to a control group, individuals with a diagnosis of an eating disorder produced fewer specific future events, although the level of episodic specificity produced for future events was not affected [20]. Our findings are somewhat surprising; as the acAN group were less able to produce specific details of memories, it would be expected that EFT specificity would also be reduced, especially given that autobiographical memory specificity predicted EFT specificity in all groups, including acAN. However, the acAN sample reported less positive EFTs, aligning with previous findings [21].

Whilst there is significant neurobiological and phenomenological overlap between autobiographical memories and EFTs [63], there are contrasting features, such as EFTs being more goal-directed, less vivid and more likely to be simulated in the third-person perspective rather than first-person [64–66]. These phenomenological differences may explain why individuals with acute AN are able to generate specific future thoughts but have impaired autobiographical memory. Individuals with AN may be goal-directed during the early stages of AN (i.e. weight loss behaviours), albeit behaviours are thought to transfer to become more habitual as the illness becomes more entrenched [67]. Additionally, individuals with AN may be better at delaying rewards, with some studies exemplifying lower scores in delay discounting tasks in individuals with acute AN [68–70], although no studies have found alterations in individuals recovered from the disorder [71, 72]. Indeed it has been found that the ability to imagine the future reduces the tendency for delay discounting [73], exemplifying a link between the two. Therefore, the goal-directed, driven nature of AN and ability to delay reward may counteract the impact of comorbid depression on EFT production, which should be explored in future research.

Notably, our acAN sample performed well on the neuropsychological domains of working memory, cognitive flexibility and verbal fluency, indicating that the results are largely independent of difficulties in other related cognitive processes. Previous studies have found that verbal fluency is often intact in AN [8, 74], although other studies have found that working memory, as well as cognitive flexibility (as measured by the WCST) is impaired [75]. There are several versions of the WCST available, differing in task length (e.g. 64 or 128 cards) and route of administration (e.g. computerised, pen and paper), and variations may explain our differing results. Moreover, there are other indices of cognitive flexibility that may have been impaired in our group of individuals with, or remitted from, AN (e.g. task switching; [4, 5]), which are distinct from the index of cognitive flexibility traditionally measured by the WCST (i.e. perseverance).

### Clinical implications

Problems with retrieving specific details of autobiographical memories may disrupt the formation of a self-narrative in individuals with AN, in understanding and placing in context the events that led up to the individual’s current reality and perceiving ones that may be expected in the future. An understanding of the personal narrative is important for the formation of one’s identity [76], which is a key feature of AN recovery and is incorporated into first-line treatments for AN such as the Maudsley Model of Anorexia Treatment for Adults (MANTRA; [77]). Moreover, the difficulties in retrieving specific autobiographical memories may mean that individuals with AN struggle to understand or learn from their past experience, or have difficulty in using their own experience to infer the behaviours of others [78], as well as to connect with others [79].

Lastly, whilst individuals with AN were able to imagine a future, they were less positive about events in the future, which may be linked to a loss of hope and an ambivalence to engage in recovery-focused goal-setting and future thinking. Treatments that target the comorbid depressive symptomatology in people with acute AN, which may interfere with the retrieval of autobiographical memories and obscure thinking positively about the future, are particularly important. However, standard treatments for depression (e.g. antidepressants) are generally ineffective in individuals with AN [80], thus novel treatments to target depressive symptomatology (e.g. [81]), if successful, may improve autobiographical memory retrieval and future thinking in AN. Based on the results of our study, psychotherapeutic strategies that focus on reconsolidating negative memories (e.g. imagery rescripting) and optimising the simulation of positive future thoughts, that have preliminary evidence in individuals with major depressive disorder [82, 83], could be considered as future clinical research avenues.

### Strengths and limitations of the present study

There were several novel aspects to this study. For example, to the authors’ knowledge, this is the first study looking at the relationship between autobiographical memory and EFT abilities in individuals with acAN; the first study investigating the impact of comorbid depressive symptomatology on EFT abilities in individuals with acAN; the first study using disorder-related cues to prime EFT production; and the first study looking at autobiographical memory and EFT abilities in individuals recovered from AN.

However, there are several limitations relating to the remote nature of this study due to the ongoing COVID-19 pandemic. Firstly, the remote administration of the tasks means that the environment in which participants completed the tasks was not controlled. However, basic features of the optimal environment for the administration of cognitive tasks, and the importance of such an environment, was reiterated to participants before commencement of the tasks and during a brief phone call. Moreover, we were able to ascertain to an extent whether people concentrated on the task, through looking at response rates, reaction times and the Gorilla interface, which allows you to monitor participant progress. The remote nature of this study has benefits, since participants took part in their natural environments, which maximised the inclusion of participants across a wider geographical span (within the United Kingdom) and was likely to be less stressful for participants. However, weight and diagnosis was also self-reported and psychiatric comorbidities were not verified using a standardised diagnostic interview, albeit diagnoses were verified verbally during a phone call where the participants’ history of clinical care was taken, and the EDE-Q was administered to verify the clinical significance of their eating disorder. Individuals with AN are usually weighed frequently by their primary care provider or specialist services, and there is evidence to suggest that they tend to give reliable estimates [84, 85]. However people remitted from AN and controls may underestimate their BMI, driven by higher subjective estimates of height [86]. Therefore, the estimated BMI of the recAN and control group may be slightly lower than objective measurements. Therefore, we did not include BMI in our main regression analyses. Despite remote task administration, the findings were largely similar to previous studies conducted in-person (e.g. [59]).

### Conclusions

This study aimed to examine the performance on, and relationship between, autobiographical memory and episodic future thinking tasks in individuals with acAN and individuals recovered from AN. Individuals with acAN showed generalised problems towards retrieving specific details of autobiographical memories and were less positive when constructing EFTs, although surprisingly the ability to simulate specific details of EFTs was intact in acAN. People recovered from AN were intact in most aspects of memory retrieval and EFT construction, although they reported greater difficulty in recalling autobiographical memories. Overall, it appears that difficulties with autobiographical memory retrieval and representations, and the construction of EFTs, is a state-related difficulty associated with acAN that is at least partially associated with comorbid depressive symptomatology. This may interfere with their ability to form a personal self-narrative and identity, and their ability to maintain hope for a future without AN.

## Supplementary Information

**Additional file 1.** Supplementary data and analyses.

## Data Availability

The datasets used in the current study are available from the corresponding author on reasonable request.

## Abbreviations

AcAN: Acute anorexia nervosa
AMT: Autobiographical memory test
AN: Anorexia nervosa
ANCOVA: Analysis of covariance
ANOVA: Analysis of variance
BMI: Body Mass Index
DASS: Depression, Anxiety and Stress Scale
EDE-Q: Eating Disorder Examination-Questionnaire
EFT: Episodic future thinking
EFT-T: Episodic future thinking task
GAD-7: Generalised Anxiety Disorder Assessment
HC: Healthy controls
M: Mean
Rec-AN: Recovered from AN
SD: Standard deviation
VAS: Visual Analogue Scale
WCST: Wisconsin Card Sorting Test
