# Supplementary material for: Difficulties in retrieving specific details of autobiographical memories and imagining positive future events in individuals with acute but not remitted anorexia nervosa
Source: J Eat Disord. 2022 Nov 18;10:172. doi: 10.1186/s40337-022-00684-w (PMC9675114; doi:10.1186/s40337-022-00684-w)
Supplement: Supplementary file 1 — Additional file 1. Supplementary data and analyses. [file 40337_2022_684_MOESM1_ESM.docx]

**Additional file 1**

“Difficulties in retrieving positive autobiographical memories and imagining positive future events in anorexia nervosa”

Table S1. Between-group differences in exploratory cognitive outcomes.

| Task | HC (n=35) | acAN (n=46) | recAN (n=40) | F-value (df) | *p*-value (Cohen’s *d*) | *acAN vs. HC*  *p*-value | *HC vs. recAN*  *p*-value | *acAN vs. recAN*  *p*-value |
| --- | --- | --- | --- | --- | --- | --- | --- | --- |
| Word fluency (total words) | 73.2±21.0 | 81.7±21.8 | 72.6±22.2 | 2.64(2,116) | 0.076 (0.43) | 0.068 | 0.875 | 0.043* |
| Digit span forwards | 6.7±1.3 | 6.9±1.0 | 6.8±1.3 | 0.37(2,115) | 0.695 (0.16) | 0.395 | 0.628 | 0.725 |
| Digit span backwards | 5.9±1.7 | 5.8±2.2 | 6.0±1.5 | 0.05(2,115) | 0.951 (0.06) | 0.804 | 0.978 | 0.777 |
| *Wisconsin card sorting task* | | | | | |  |  |  |
| **General performance** | | | | | |  |  |  |
| Number of trials administered | 31.5±18.9 | 34.4±19.5 | 39.1±26.6 | 0.78(2,111) | 0.459 (0.24) | 0.453 | 0.215 | 0.587 |
| Total correct responses | 70.8±12.9 | 72.0±10.2 | 72.0±11.9 | 0.20(2,111) | 0.817 (0.13) | 0.528 | 0.775 | 0.528 |
| Total response errors | 27.5±22.0 | 25.5±19.9 | 33.0±22.2 | 2.11(2,111) | 0.126 (0.39) | 0.497 | 0.207 | 0.044 |
| Total categories completed | 5.3±1.6 | 5.3±1.2 | 4.9±1.7 | 1.94(2,111) | 0.149 (0.38) | 0.652 | 0.172 | 0.059 |
| **Perseveration** | | | | | |  |  |  |
| Perseverative responses (%) | 11.1±3.8 | 12.9±5.6 | 11.4±5.5 | 1.54(2,111) | 0.219 (0.33) | 0.102 | 0.706 | 0.201 |
| Perseverative errors (%) | 7.9±2.8 | 8.6±3.8 | 7.9±4.2 | 0.56(2,111) | 0.575 (0.20) | 0.317 | 0.789 | 0.458 |
| Non-perseverative errors (%) | 17.6±17.0 | 14.8±14.1 | 21.2±17.3 | 2.36(2,111) | 0.099 (0.41) | 0.294 | 0.301 | 0.032* |
| **Conceptual ability** | | | | | |  |  |  |
| Trials to complete first category | 16.7±20.1 | 12.9±4.3 | 17.7±20.1 | 3.83(2,111) | 0.025* (0.53) | 0.051 | 0.566 | 0.010* |
| **Response consistency** | | | | | |  |  |  |
| Failure to maintain set | 0.8±1.2 | 0.8±1.3 | 1.1±1.3 | 0.54(2,111) | 0.584 (0.20) | 0.934 | 0.408 | 0.339 |
| Learning to learn | -0.5±4.1 | -0.9±3.5 | 0.5±3.5 | 3.37(2,111) | 0.038* (0.49) | 0.409 | 0.105 | 0.012* |

**Significant at the *p<*0.01 threshold*, **Significant at the *p<*0.05 threshold*.* All analyses were run with age and years of education as covariates and *p*-values for post-hoc group comparisons were Bonferroni adjusted. acAN = acute anorexia nervosa; HC = healthy controls; rec-AN = recovered anorexia nervosa.

Table S2. Effect of covariates on within-subjects effect of valence

|  | **Age** | | **Education** | | |
| --- | --- | --- | --- | --- | --- |
|  | F-value (*df*=4,232) | *p*-value | F-value (*df*=4,232) | *p*-value | |
| *Autobiographical Memory Task* | | | | |  |
| Specificity^a^ | 4.87 | 0.008** (0.41) | 0.10 | 0.902 (0.06) | |
| Detailedness^b^ | 2.00 | 0.138 (0.26) | 0.44 | 0.647 (0.13) | |
| Difficulty to remember^b^ | 0.06 | 0.944 (0.06) | 0.15 | 0.863 (0.06) | |
| Realistic^b^ | 0.58 | 0.559 (0.14) | 0.81 | 0.444 (0.17) | |
| Positivity^b^ | 1.90 | 0.152 (0.26) | 0.65 | 0.526 (0.16) | |
| Vividness^b^ | 0.56 | 0.571 (0.14) | 1.38 | 0.254 (0.22) | |
| *Episodic Future Thinking Task* | | | | |  |
| Specificity^a^ | 1.11 | 0.332 (0.19) | 0.02 | 0.983 (0.06) | |
| Detailedness^b^ | 0.39 | 0.680 (0.11) | 0.36 | 0.700 (0.11) | |
| Difficulty to imagine^b^ | 0.70 | 0.497 (0.16) | 0.56 | 0.571 (0.14) | |
| Realistic^b^ | 0.40 | 0.669 (0.11) | 0.94 | 0.394 (0.18) | |
| Positivity^b^ | 0.13 | 0.880 (0.06) | 1.70 | 0.184 (0.24) | |
| Vividness^b^ | 0.24 | 0.713 (0.09) | 1.88 | 0.168 (0.26) | |

*Notes.* **Significant at the *p<*0.01 threshold*, **Significant at the *p<*0.05 threshold. ^a^experimenter rated, ^b^participant rated.

Table S3. Effect of covariates on between-subjects effects

|  | **Age** | | **Education** | | |
| --- | --- | --- | --- | --- | --- |
|  | F-value (*df*=2,232) | *p*-value | F-value (*df*=2,232) | *p*-value | |
| *Autobiographical Memory Task* | | | | |  |
| Specificity^a^ | 3.09 | 0.082 (0.33) | 0.49 | 0.487 (0.13) | |
| Detailedness^b^ | 2.87 | 0.093 (0.31) | 3.94 | 0.050* (0.37) | |
| Difficulty to remember^b^ | 0.28 | 0.597 (0.09) | 2.11 | 0.149 (0.27) | |
| Realistic^b^ | 0.99 | 0.321 (0.18) | 3.11 | 0.080 (0.33) | |
| Positivity^b^ | 0.19 | 0.661 (0.09) | 0.19 | 0.664 (0.09) | |
| Vividness^b^ | 0.25 | 0.616 (0.09) | 0.11 | 0.745 (0.06) | |
| *Episodic Future Thinking Task* | | | | |  |
| Specificity^a^ | 6.14 | 0.015* (0.46) | 1.08 | 0.302 (0.19) | |
| Detailedness^b^ | 0.08 | 0.784 (0.06) | 2.87 | 0.093 (0.31) | |
| Difficulty to imagine^b^ | 4.23 | 0.042* (0.38) | 2.21 | 0.140 (0.28) | |
| Realistic^b^ | 0.67 | 0.414 (0.16) | 0.98 | 0.324 (0.18) | |
| Positivity^b^ | 0.50 | 0.483 (0.13) | 0.01 | 0.985 (0.06) | |
| Vividness^b^ | 0.06 | 0.804 (0.06) | 0.04 | 0.849 (0.06) | |

*Notes.* **Significant at the *p<*0.01 threshold*, **Significant at the *p<*0.05 threshold. ^a^experimenter rated, ^b^participant rated.

Table S4. Supplementary ANCOVA models controlling for ethnicity, DASS-Depression scores and average sleep over the past 3 days for autobiographical memory task outcomes.

|  | Autobiographical Memory Task | | | | | | | | |
| --- | --- | --- | --- | --- | --- | --- | --- | --- | --- |
|  | **Valence** | | | **Group** | | | **Valence x Group** | | |
|  | F-value | *df* | *p*-value (Cohen’s *d*) | F-value | *df* | *p*-value (Cohen’s *d*) | F-value | *df* | *p*-value (Cohen’s *d*) |
| *Controlling for ethnicity* | | | | | | | | | |
| Specificity^a^ | 0.71 | 2,228 | 0.491 (0.16) | 3.24 | 2,114 | 0.043* (0.48) | 1.42 | 2,228 | 0.228 (0.31) |
| Difficulty to remember^b^ | 0.45 | 2,228 | 0.637 (0.13) | 5.92 | 2,114 | 0.004** (0.64) | 1.77 | 2,228 | 0.136 (0.35) |
| Positivity^b^ | 4.05 | 2,228 | 0.019* (0.38) | 5.83 | 2,114 | 0.004** (0.64) | 2.11 | 2,228 | 0.080 (0.39) |
| Vividness^b^ | 2.72 | 2,228 | 0.068 (0.31) | 2.09 | 2,114 | 0.129 (0.38) | 2.26 | 2,228 | 0.063 (0.40) |
| *Controlling for DASS-Depression* | | | | | | | | | |
| Specificity^a^ | 0.50 | 2,230 | 0.609 (0.13) | 1.63 | 2,115 | 0.201 (0.33) | 0.81 | 2,230 | 0.521 (0.24) |
| Difficulty to remember^b^ | 0.16 | 2,230 | 0.853 (0.06) | 2.92 | 2,115 | 0.058 (0.45) | 1.24 | 2,230 | 0.293 (0.29) |
| Positivity^b^ | 6.09 | 2,230 | 0.003** (0.46) | 4.03 | 2,115 | 0.020* (0.53) | 0.84 | 2,230 | 0.499 (0.24) |
| Vividness^b^ | 1.66 | 2,230 | 0.193 (0.24) | 0.45 | 2,115 | 0.640 (0.18) | 1.53 | 2,230 | 0.193 (0.33) |
| *Controlling for average sleep over past 3 days* | | | | | | | | | |
| Specificity^a^ | 1.01 | 2,228 | 0.364 (0.19) | 4.80 | 2,114 | 0.010* (0.58) | 2.56 | 2,228 | 0.039* (0.42) |
| Difficulty to remember^b^ | 0.24 | 2,228 | 0.790 (0.09) | 3.57 | 2,114 | 0.031* (0.50) | 1.88 | 2,228 | 0.114 (0.36) |
| Positivity^b^ | 2.35 | 2,228 | 0.098 (0.29) | 5.26 | 2,114 | 0.007** (0.61) | 1.68 | 2,228 | 0.155 (0.35) |
| Vividness^b^ | 1.44 | 2,228 | 0.239 (0.22) | 1.59 | 2,114 | 0.208 (0.33) | 2.36 | 2,228 | 0.054 (0.41) |

*Notes.* **Significant at the *p<*0.01 threshold*, **Significant at the *p<*0.05 threshold. ^a^experimenter rated, ^b^participant rated. All analyses additionally controlled for age and years of education.

Table S5. Supplementary ANCOVA models controlling for ethnicity, DASS-Depression scores and average sleep over the past 3 days for episodic future thinking task outcomes.

|  | Episodic Future Thinking Task | | | | | | | | |
| --- | --- | --- | --- | --- | --- | --- | --- | --- | --- |
|  | **Valence** | | | **Group** | | | **Valence x Group** | | |
|  | F-value | *df* | *p*-value (Cohen’s *d*) | F-value | *df* | *p*-value (Cohen’s *d*) | F-value | *df* | *p*-value (Cohen’s *d*) |
| *Controlling for ethnicity* | | | | | | | | | |
| Positivity^b^ | 2.87 | 2,228 | 0.059 (0.32) | 5.85 | 2,114 | 0.004** (0.64) | 3.92 | 2,228 | 0.004** (0.52) |
| *Controlling for DASS-Depression* | | | | | | | | | |
| Positivity^b^ | 2.84 | 2,230 | 0.061 (0.31) | 4.40 | 2,115 | 0.014* (0.55) | 2.33 | 2,230 | 0.057 (0.40) |
| *Controlling for average sleep over past 3 days* | | | | | | | | | |
| Positivity^b^ | 0.61 | 2,228 | 0.544 (0.14) | 6.33 | 2,114 | 0.002** (0.67) | 3.57 | 2,228 | 0.008** (0.50) |

*Notes.* **Significant at the *p<*0.01 threshold*, **Significant at the *p<*0.05 threshold. ^a^experimenter rated, ^b^participant rated. All analyses additionally controlled for age and years of education.

Table S6. Results of linear regression models investigating the effect of DASS-Depression scores and control regressors (age and years of education) on autobiographical memory test and episodic future thinking task outcomes in the acute anorexia nervosa group.

| Measure | Adjusted R^2^ (SE) | F-value  (*df*=3,45) | *p*-value for overall model | Independent Variable | Unstandardised Beta (SE) | *ß* | T | *p*-value for regressor |
| --- | --- | --- | --- | --- | --- | --- | --- | --- |
| *Autobiographical Memory Task* | | | | | | | | |
| Specificity^a^ | 0.096 (0.57) | 2.59 | 0.066 | (Constant) | 1.715 (0.68) |  | 2.533 | 0.015* |
|  |  |  |  | DASS-Depression | 0.006 (0.01) | 0.119 | 0.797 | 0.430 |
|  |  |  |  | Age | 0.026 (0.013) | 0.316 | 2.075 | 0.044* |
|  |  |  |  | Years of education | -0.055 (0.039) | -0.207 | -1.420 | 0.163 |
| Detailedness^b^ | 0.001 (1.03) | 1.02 | 0.393 | (Constant) | 5.104 (1.230) |  | 4.151 | <0.001** |
|  |  |  |  | DASS-Depression | -0.020 (0.013) | -0.241 | -1.534 | 0.133 |
|  |  |  |  | Age | 0.028 (0.023) | 0.199 | 1.243 | 0.221 |
|  |  |  |  | Years of education | -0.043 (0.071) | -0.092 | -0.600 | 0.552 |
| Difficulty to remember^b^ | -0.010 (0.85) | 0.85 | 0.476 | (Constant) | 1.280 (1.016) |  | 1.260 | 0.215 |
|  |  |  |  | DASS-Depression | 0.011 (0.011) | 0.157 | 0.995 | 0.326 |
|  |  |  |  | Age | -0.007 (0.019) | -0.056 | -0.347 | 0.730 |
|  |  |  |  | Years of education | 0.079 (0.059) | 0.207 | 1.345 | 0.186 |
| Realistic^b^ | -0.065 (0.87) | 0.08 | 0.968 | (Constant) | 5.937 (1.043) |  | 5.695 | <0.001** |
|  |  |  |  | DASS-Depression | 0.002 (0.011) | 0.035 | 0.217 | 0.829 |
|  |  |  |  | Age | 0.003 (0.019) | 0.029 | 0.177 | 0.860 |
|  |  |  |  | Years of education | -0.023 (0.060) | -0.061 | -0.385 | 0.702 |
| Positivity^b^ | -0.052 (0.74) | 0.26 | 0.855 | (Constant) | 3.843 (0.883) |  | 4.351 | <0.001** |
|  |  |  |  | DASS-Depression | -0.001 (0.009) | -0.011 | -0.065 | 0.948 |
|  |  |  |  | Age | 0.012 (0.016) | 0.123 | 0.750 | 0.457 |
|  |  |  |  | Years of education | -0.029 (0.051) | -0.090 | -0.573 | 0.570 |
| Vividness^b^ | -0.024 (1.14) | 0.65 | 0.587 | (Constant) | 4.294 (1.361) |  | 3.155 | 0.003** |
|  |  |  |  | DASS-Depression | -0.011 (0.015) | -0.125 | -0.785 | 0.437 |
|  |  |  |  | Age | -0.011 (0.025) | -0.070 | -0.435 | 0.666 |
|  |  |  |  | Years of education | 0.073 (0.079) | 0.143 | 0.924 | 0.361 |
| *Episodic Future Thinking Task* | | | | | | | | |
| Specificity^a^ | 0.189 (0.71) | 4.49 | 0.008** | (Constant) | 2.333 (0.849) |  | 2.747 | 0.009** |
|  |  |  |  | DASS-Depression | 0.019 (0.009) | 0.298 | 2.103 | 0.042* |
|  |  |  |  | Age | 0.026 (0.016) | 0.235 | 1.626 | 0.111 |
|  |  |  |  | Years of education | -0.096 (0.049) | -0.271 | -1.961 | 0.057 |
| Detailedness^b^ | -0.013 (1.05) | 0.81 | 0.495 | (Constant) | 3.998 (1.250) |  | 3.198 | 0.003** |
|  |  |  |  | DASS-Depression | -0.019 (0.013) | -0.225 | -1.423 | 0.162 |
|  |  |  |  | Age | 0.005 (0.023) | 0.038 | 0.234 | 0.816 |
|  |  |  |  | Years of education | 0.032 (0.072) | 0.069 | 0.444 | 0.659 |
| Difficulty to remember^b^ | -0.024 (1.06) | 0.64 | 0.591 | (Constant) | 2.476 (1.266) |  | 1.956 | 0.057 |
|  |  |  |  | DASS-Depression | 0.009 (0.014) | 0.108 | 0.681 | 0.500 |
|  |  |  |  | Age | 0.022 (0.024) | 0.151 | 0.933 | 0.356 |
|  |  |  |  | Years of education | -0.004 (0.073) | -0.008 | -0.049 | 0.961 |
| Realistic^b^ | -0.049 (0.97) | 0.30 | 0.827 | (Constant) | 5.726 (1.155) |  | 4.959 | <0.001** |
|  |  |  |  | DASS-Depression | 0.001 (0.012) | 0.010 | 0.061 | 0.952 |
|  |  |  |  | Age | -0.016 (0.021) | -0.122 | -0.742 | 0.462 |
|  |  |  |  | Years of education | -0.025 (0.067) | -0.060 | -0.382 | 0.704 |
| Positivity^b^ | 0.002 (0.96) | 1.03 | 0.391 | (Constant) | 3.641 (1.144) |  | 3.183 | 0.003** |
|  |  |  |  | DASS-Depression | -0.016 (0.012) | -0.200 | -1.271 | 0.211 |
|  |  |  |  | Age | -0.005 (0.021) | -0.039 | -0.241 | 0.810 |
|  |  |  |  | Years of education | 0.063 (0.066) | 0.145 | 0.948 | 0.349 |
| Vividness^b^ | 0.056 (1.03) | 1.90 | 0.145 | (Constant) | 3.096 (1.230) |  | 2.517 | 0.016* |
|  |  |  |  | DASS-Depression | -0.019 (0.013) | -0.218 | -1.429 | 0.160 |
|  |  |  |  | Age | -0.012 (0.023) | -0.083 | -0.536 | 0.594 |
|  |  |  |  | Years of education | 0.112 (0.071) | 0.236 | 1.583 | 0.121 |

*Notes.* ^a^experimenter rated, ^b^participant rated, **significant at *p*<0.01, *significant at *p*<0.05. All analyses were run with age and years of education as covariates. *ß* = standardised beta; DASS = Depression, Anxiety and Stress Scale; SE = standard error.
